# Local Hydrogen Concentration and Distribution in Pd Nanoparticles: An In Situ STEM‐EELS Approach

**DOI:** 10.1002/smll.202407092

**Published:** 2024-12-08

**Authors:** Svetlana Korneychuk, Stefan Wagner, Darius Rohleder, Philipp Vana, Astrid Pundt

**Affiliations:** ^1^ Institute for Applied Materials – Materials Science and Engineering (IAM‐WK) Karlsruhe Institute of Technology Engelbert‐Arnold‐Straße 4 76131 Karlsruhe Germany; ^2^ Institute of Nanotechnology Karlsruhe Institute of Technology Hermann‐von‐Helmholtz‐Platz 1 76344 Eggenstein‐Leopoldshafen Germany; ^3^ Karlsruhe Nano Micro Facility (KNMFi) Karlsruhe Institute of Technology Herrmann‐von‐Helmholtz‐Platz 1 76344 Eggenstein‐Leopoldshafen Germany; ^4^ Institute of Physical Chemistry Georg‐August‐University Göttingen Tammannstr. 6 37077 Göttingen Germany; ^5^ Wöhler Research Institute for Sustainable Chemistry (WISCh) Georg‐August‐University Göttingen Tammannstr. 2 37077 Göttingen Germany

**Keywords:** hydrogen, nanoparticles, STEM‐EELS, strain

## Abstract

Local detection of hydrogen concentration in metals is of central importance for many areas of hydrogen technology, such as hydrogen storage, detection, catalysis, and hydrogen embrittlement. A novel approach to measure the hydrogen concentration in a model system consisting of cubic palladium nanoparticles (Pd NPs), with a lateral resolution down to 4 nm is demonstrated. By measuring the shift of the Pd bulk plasmon peak with scanning transmission electron microscopy (STEM) combined with energy electron loss spectroscopy (EELS) during in situ hydrogen gas loading and unloading, local detection of the hydrogen concentration is achieved in TEM. With this method, concentration changes inside the NPs at various stages of hydrogenation/dehydrogenation are observed with nanometer resolution. The versatility of in situ TEM allows to link together microstructure, hydrogen concentration, and local strain, opening up a new chapter in hydrogen research.

## Graphical abstract

Local detection of hydrogen concentration in metals is of central importance for many areas of hydrogen technology. By combining electron loss spectroscopy (EELS) with in situ gas TEM, the local hydrogen concentration can be measured in a model system consisting of cubic palladium nanoparticles (Pd NPs), with a lateral resolution down to 4 nm. The versatility of in situ TEM allows to link together microstructure, local hydrogen concentration, and local strain, opening up a new chapter in hydrogen research.

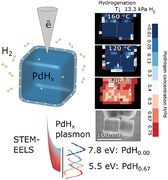

## 1 Introduction

In the transition to a sustainable economy, hydrogen can play a leading role as one of the key renewable energy carriers. In this context, the knowledge of the local hydrogen distribution and concentration is crucial for improving hydrogen storage materials, enhancing hydrogen sensing and early detection, refining processes in catalysis, and advancing the understanding of hydrogen embrittlement mechanisms.^[^ ^1^, ^2^, ^3^, ^4^, ^5^, ^6^, ^7^, ^8^, ^9^, ^10^ ^]^ However, local measurement of hydrogen concentrations remains a challenge to this day. Existing methods for observing hydrogen in materials lack either lateral resolution or in situ capabilities (seeSupporting Information).^[^ ^11^, ^12^, ^13^, ^14^, ^15^ ^]^

Understanding the distribution and local concentration of hydrogen is of particular interest in nanomaterials. Compared to bulk systems, the distribution of hydrogen in the metal lattice and the stability of hydride phases can be different in nanosized systems,^[^ ^16^, ^17^, ^18^, ^19^, ^20^, ^21^, ^22^, ^23^, ^24^, ^25^ ^]^ such as thin films and nanoparticles (NPs). High surface‐to‐volume ratio, special microstructure, or elastic constraints resulting from stabilizers supporting nanosystems are just a few contributing factors. Hydrogen absorption is most complex in NPs due to the influence of numerous interfering factors, including their structure and shape, the presence or absence of defects, and strain. Phase transformations in NPs are still not very well understood at the nanoscale. Experimental studies on a large scale, along with theoretical considerations^[^ ^26^, ^27^, ^28^, ^29^ ^]^ on phase transformations, phase stability, and hydrogen distribution in nanosystems, require experimental confirmation at the local scale. According to studies by Yamauchi et al.,^[^ ^30^ ^]^ in Pd NPs larger than 10 nm the chemical potentials for hydrogen absorption and desorption are expected to resemble bulk behavior. However, for clusters smaller than 4.6 nm, structural changes occur,^[^ ^18^, ^31^, ^32^ ^]^ affecting hydrogen uptake. Even for larger NPs, any preexisting elastic stresses due to, e.g., strong adhesion to a substrate will alter the chemical potential^[^ ^33^, ^34^, ^35^ ^]^ and, hence, change pressure‐concentration isotherms. In this study, we investigate the hydrogen absorption and desorption in cubic, defect‐free Pd NPs with an average size of 67 nm and preexisting elastic strain. According to Mütschele and Kirchheim,^[^ ^36^ ^]^ or Sachs et al.,^[^ ^32^ ^]^ the hydride formation in nanomaterials is expected to happen only on the inner bulk‐like lattice sites. Mütschele et al.,^[^ ^36^ ^]^ who determined the phase boundaries of nanocrystalline PdH_x_, distinguished between bulk and grain boundary regions and interpreted the observed narrowing of the miscibility gap with an enhanced hydrogen solubility at the grain boundaries compared to the solid solution α‐phase. Sachs et al.,^[^ ^32^ ^]^ applied this model to PdH_x_ NPs by a distinction between surface/subsurface (shell) and bulk regions. They assumed that the shell region, similar to grain boundaries, does not transform into the hydride phase and determined a hydrogen concentration of up to 0.44 H/Pd in the shell. This concentration is an intermediate value between those of bulk solid solution α‐ and hydride α’‐ phase. In their interpretation, Sachs et al. assumed a shell thickness of two atomic layers. Direct verification of these results requires the local measurement of the hydrogen concentration at the surface of Pd NPs. Then, the presence of intermediate hydrogen concentrations and the proposed absence of hydride formation at the surface can be assessed, clarifying the thermodynamic properties of the surface in comparison to the core of the NPs. This is the main aim of the present manuscript.

Transmission electron microscopy (TEM) is an ideal tool to access materials properties at the nanoscale. Baldi et al.,^[^ ^1^ ^]^ demonstrated that a shift of the Pd bulk plasmon energy from 7.7 to ≈5.5 eV upon hydrogenation can be observed with scanning transmission electron microscopy (STEM) combined with electron energy loss spectroscopy (EELS) in an environmental TEM for cubic NPs of 13–29 nm in size. The shift happens due to the change in the electronic density of states in PdH_x_ as it transforms from a Pd solid solution to a Pd hydride. This work showed sharp α‐ to α΄‐ phase transition and large hysteresis in the single crystalline cubic NPs upon cyclic hydrogen loading and unloading. Subsequent research by the same group of Dionne et al., examined the phase transition in the Pd NPs in greater detail, revealing that the nucleation of the hydride phase starts from the corners and forms a (100) phase front that grows across the NP.^[^ ^37^ ^]^ Further work demonstrated that crystallographic defects and strain‐induced deformations in the Pd NPs influence the hydrogen uptake.^[^ ^38^, ^39^ ^]^

In the present manuscript, we extend the approach of Baldi et al. to a quantification of the local hydrogen concentrations by measuring the shift of the Pd bulk plasmon upon sequential hydrogen loading and unloading of the Pd NPs at the local scale. We demonstrate here that combining TEM with in situ gas loading enables nanometer‐scale observation of the hydrogen interaction with the model cubic Pd NPs in real time. This method facilitates the assessment of fundamental aspects of hydrogen sorption and hydride formation in Pd NPs, allowing us to verify the model assumptions outlined above. Key areas of interest include the local hydrogen concentration, the transformation behavior of surfaces, subsurfaces, and interfaces, as well as the related phase transformation pressures, the width, and the position of the miscibility gap at the concentration axis. The versatility of in situ TEM enables the simultaneous measurement of strain and hydrogen concentration under identical conditions. This allows us to address the topic of hydrogenation being affected by preexisting strain in the NPs.

## 2 Experimental Section

**Figure 1:**
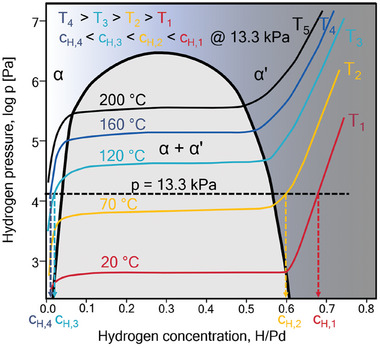
Sketch of pressure‐concentration isotherms for bulk PdH_x_ at different temperatures (see data of Manchester et al.^[^ ^40^ ^]^). The bold black curve marks the region of the two‐phase field where α‐ and α’‐ phases coexist and the isotherms possess a plateau.

Hydrogen occupies octahedral sites in the undistorted Pd metal lattice. When the hydrogen concentration is low, the PdH_x_ system stays in the solid solution phase until the α΄‐hydride‐phase starts to form at the solid solution limit. The phase change from α‐solid solution to α’‐ hydride phase in the NP PdH_x_ system is reversible. The transformation can be achieved by changing the hydrogen pressure and/or the temperature, as shown in the isotherm sketch in **Figure** **1** for the bulk PdH_x_ system. In this work, the phase transformation is studied by changing the temperature while maintaining a constant hydrogen pressure, as illustrated in Figure 1. By changing the temperature while remaining on the same horizontal pressure line, the sample condition crosses different isotherms and thereby switches between the α‐solid solution phase at high temperatures and the α’‐ hydride phase at low temperatures. The corresponding solubility limits depend on the temperature, as indicated by the turning points of the isotherms. In the PdH_x_ system, the hydrogen concentration is inversely related to the temperature at a constant pressure: it increases with the temperature decline, as illustrated in Figure 1. For example, at a constant pressure of 13.3 kPa, the PdH_x_ system will transform from the solid solution α phase to the α΄‐hydride, if the temperature decreases from 120 to 70 °C. Conversely, with an increase in pressure, the transformation from α to α΄ phase occurs at higher temperatures. For instance, at 100 kPa, the system forms the α΄‐phase when it is cooled below 160 °C.^[^ ^40^ ^]^

Hydrogen uptake and release in cubic Pd NPs^[^ ^41^, ^42^ ^]^ is studied by in situ TEM with the in situ gas cell Atmosphere AX from Protochips in a ThermoFisher Scientific Themis Z electron microscope. In this setup the sample was tightly sealed between two micro‐electromechanical systems chips and analyzed through electron‐transparent SiN windows. The NPs are stable below 200 °C. To observe the Pd hydride phase formation within this temperature range, hydrogen pressures up to half an atmosphere were applied. STEM‐EELS maps of isobaric measurements at *p*  =  13.3 kPa and *p* = 26.7 kPa hydrogen gas pressure were acquired at different temperatures. The temperature was ramped down in steps of 20 °C, exemplarily starting at 160 °C and ending at 60 °C. To observe dehydrogenation, the temperature was then increased again from 60 or 80 °C back to 160 °C with the same increments, completing a full hydrogenation/dehydrogenation cycle. The EELS maps were collected after keeping the NPs at each temperature for 5 min. The electron beam was blanked between acquisitions. In this study, a significant influence of the electron beam on the hydrogenation/dehydrogenation process of the Pd NPs was not observed. In particular, PdH_x_ NPs did not noticeably release or absorb hydrogen upon exposure to the electron beam.

More detailed information about possible effects of the electron beam, along with the experimental setup and EELS simulations, is given in the Supporting Information.^[^ ^43^, ^44^ ^]^ It includes an overview of the established methods for the measurement of hydrogen concentration, a description of the cubic Pd NPs sample preparation, simulations of the PdH_x_ plasmon shift, studies of the influence of the NP shape on the simulated PdH_x_ plasmon shift, experimental details on the measurement of the Pd plasmon shift with TEM, measurements of strain in PdH_x_ NPs with TEM^[^ ^45^, ^46^ ^]^ on different substrates, and general aspects of strain effects in metal hydrogen systems.

## 3 Local Measurement of Concentration: Explanation Based on the Plasmon Bulk Shift

STEM‐EELS can provide lateral resolution of 3–4 nm^[^ ^47^ ^]^ (see Supporting Information) for hydrogen detection together with in situ capabilities and non‐destructive measurement of the hydrogenation of the NPs.

In the present experiment, the hydrogen content is detected by measuring the Pd bulk plasmon position, which depends on the local hydrogen concentration. The shift of the bulk plasmons originates from changes in the electronic density of states in Pd during hydrogen absorption.^[^ ^48^ ^]^ As mentioned above, the bulk plasmon peak shifts upon hydrogenation from 7.7 eV for solid solution (H/Pd < 0.01) to 5.5 eV.^[^ ^1^ ^]^ The latter position corresponds to a hydrogen concentration of x = 0.67 H/Pd at 20 °C.^[^ ^49^ ^]^ Bennett et al. report that the bulk plasmon of Pd can decrease further to 4.5 eV if the hydrogen concentration reaches ≈0.8 H/Pd.^[^ ^50^ ^]^ Palm et al. showed that the experimental dielectric function of PdH_x_ changes gradually with the increase of hydrogen concentration,^[^ ^51^ ^]^ which can be utilized for hydrogen sensing.^[^ ^52^ ^]^ However, the bulk plasmon region was not measured in that study.^[^ ^51^ ^]^

**Figure 2:**
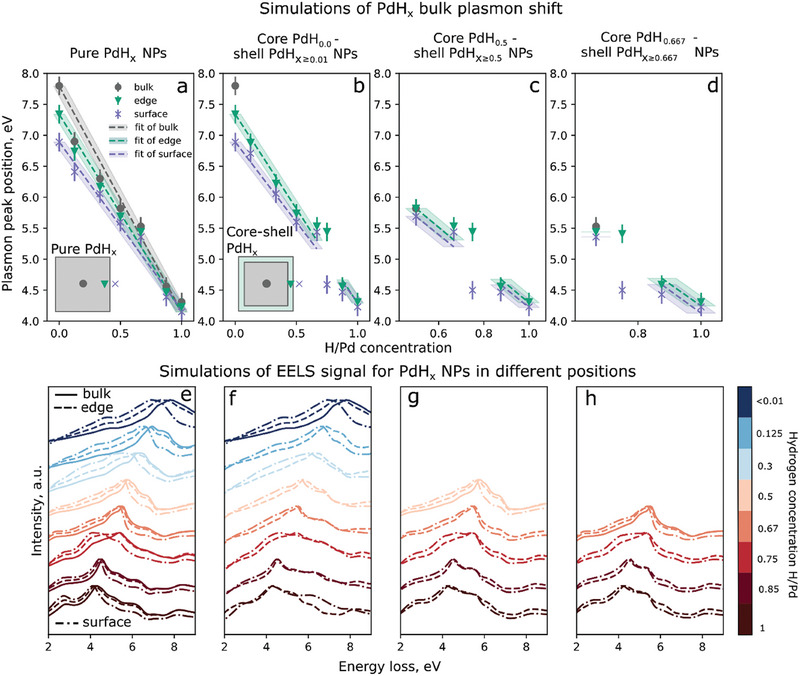
Simulated positions and spectra of the PdH_x_ plasmon for various x based on the simulated dielectric functions extracted from.^[^ ^53^ ^]^ a) Shift of the maximum of the bulk plasmon peak of pure PdH_0≤x≤1 _66 nm NPs at different positions – middle of the NP, which is similar to the bulk PdH_x_ (gray circles), edge of the NP (green triangles) and surface of the NP (violet crosses). The values in the middle position are fitted with the linear function *E*_bulk_ (eV) =  7.80 − 3.64*x*_*H*/*Pd*_ for bulk which is used later for determining the H/Pd concentration in the experimental data shown in Figure 3. Experimental error bars are added to the simulated plasmon positions to assess the capabilities and limitations of our experiment. The position of the plasmon peak can be determined with the best precision of about ±150 meV which leads to an error of ±0.05 H/Pd in the estimation of the concentration. In cases of b–d) the shift of the maximum of the bulk plasmon was simulated for the core–shell NP in the middle, at the edge (in the shell), and at the surface of the NP. b) The core concentration is below 0.01 H/Pd, and the shell concentration ranges from 0 to 1 H/Pd in the simulated NP. c) The core concentration is 0.5 H/Pd, and the shell concentration ranges from 0.5 to 1 H/Pd. d. The core concentration is 0.67 H/Pd, and the shell concentration ranges from 0.67 to 1 H/Pd. When the concentration in the core equals the concentration in the shell, the values for the pure NPs are displayed. e–h) graphs represent corresponding simulated EELS spectra for NPs in a–d) accordingly. Solid lines correspond to the spectra simulated in the middle of the NPs, dashed lines to the edge/shell, and dotted and dashed lines to the positions at the surface of the NP.

To link the energy of the Pd bulk plasmon to any specific hydrogen concentration, we use ab initio time‐dependent density‐functional theory ‐based simulations of the dielectric optical response of bulk stoichiometric and homogeneous PdH_x_, that were performed by Silkin et al.^[^ ^53^ ^]^ and Poyli et al.^[^ ^54^ ^]^ These theoretical studies showed that the peak position of the bulk plasmon of PdH_x_ shifts to lower energies almost linearly with an increase of hydrogen concentration, from 7.78 eV in pure Pd to 4.25 eV in PdH_1_. Thus, we determine the related hydrogen concentration by comparing the position of the experimental PdH_x_ plasmon peak maximum to the reference values simulated for PdH_0≤x≤1_ concentration. Reference PdH_x_ bulk plasmon EELS spectra are simulated^[^ ^55^, ^56^ ^]^ for 66 nm NP using the dielectric functions from Silkin et al.^[^ ^53^ ^]^ These spectra are presented in **Figure** **2**e for the center, the edge, and the surface of a PdH_x_ NP, with the corresponding shift of the plasmon peak position in Figure 2a. Details of the simulation are provided in the Supporting Information.

The PdH_x_ NP plasmon energy shifts almost linearly with the increase of x, as indicated in Figure 2a by the blue symbols. As a straightforward approach, we approximate the values, simulated in the center of the NP, with the linear function *E*_bulk_ (eV) =  7.80 − 3.64*x*_*H*/*Pd*_ (1). The experimentally confirmed value of 7.8 eV for pure Pd NPs is given a stronger weight in the fitting process. To achieve a more precise approximation of the plasmon shift in relation to the hydrogen concentration, more detailed fitting models are planned to be explored in future work.

At the local positions of the edges and the surface of the NPs, a redshift of the plasmon maximum is detected in the simulations (Figure 2a,e), with a similar quasi‐linear dependency on the hydrogen concentration. This shift is considered for the accurate interpretation of the hydrogen concentration at the surface of the NPs, in the experimental results. (For details also see the Supporting Information.)

In the experiment, we observe the start of the hydrogenation at the surface with a thin shell forming around the NPs. This process is discussed in detail later. This asks for an accurate separation of the real hydrogen concentration leading to a plasmon peak position shift, from the inherent redshift present at the surface of pure NPs. For this, we simulate core–shell NPs with core H/Pd concentrations of x˂0.01, 0.5, and 0.67 H/Pd, observed in the experiment, and shell concentrations ranging from 0 to 1, 0.5 to 1, and 0.67 to 1 H/Pd, correspondingly. Maximum bulk plasmon values for these three cases are presented in Figure 2b,c,d. They were extracted from the simulated spectra presented in Figure 2f,g,h. The physical thickness of the shell is considered to be ≈3 nm and the total NP diameter is 66 nm, in the simulation. This allows for reliable calculation results also in the shell. In the core of the simulated NP the maximum value of the bulk plasmon does not change with different shell concentrations (gray circles in Figure 2b,c,d). Plasmon peak positions simulated in the shell with various concentrations closely match the values in the same regions of pure NPs (simulated without shell) with the same concentration. For the accurate interpretation of hydrogen concentration in the shell, both values–at the edge of the NP marked with green triangles in Figure 2b,c,d, and at the surface of the NP marked with purple crosses in Figure 2b,c,d —are considered (see Supporting Information).

As shown in Figure 2b, in the case of a NP with 0 H/Pd in the core, the shell with a concentration of 0.125 H/Pd can already be distinguished from the redshift of the bulk plasmon at the surface of a pure Pd NP. In the last two cases with core concentrations of 0.50 and 0.67 H/Pd demonstrated in Figure 2c,d, hydride formation in the shell with higher concentration than in the core can be detected according to the simulations when the shell concentrations are 0.67 and 0.75 H/Pd, correspondingly.

The 0.75 H/Pd concentration represents a notable exception. The bulk plasmon peak at this concentration does not have a sharp maximum spreading from ≈4.2 eV to 5.3 eV, especially at the edge and surface of the NPs (Figure 2e–h). According to Silkin,^[^ ^53^ ^]^ at this concentration, the d‐band of PdH_0.75_ becomes completely occupied and goes below the Fermi level. In this condition, the contribution of interband transitions at the Fermi level in the excitation optical spectrum and in the EELS signal is drastically reduced.

In all three core–shell configurations, the concentration of 0.75 H/Pd at the surface of the NPs cannot be distinguished from 0.875 or 1 H/Pd concentration due to the flat shape of the plasmon peak at 0.75 H/Pd spreading over 1 eV. The only indication of hydride formation with a concentration of 0.8 H/Pd and higher would be a sharp bulk plasmon peak. However, in the experiment, no sharp peaks at 4.5 eV and lower are observed, and, therefore, all the peak positions below 5.3 eV are attributed to 0.75 H/Pd concentration.

## 4 Experimental EELS Measurement of Hydrogen Concentration

**Figure 3:**
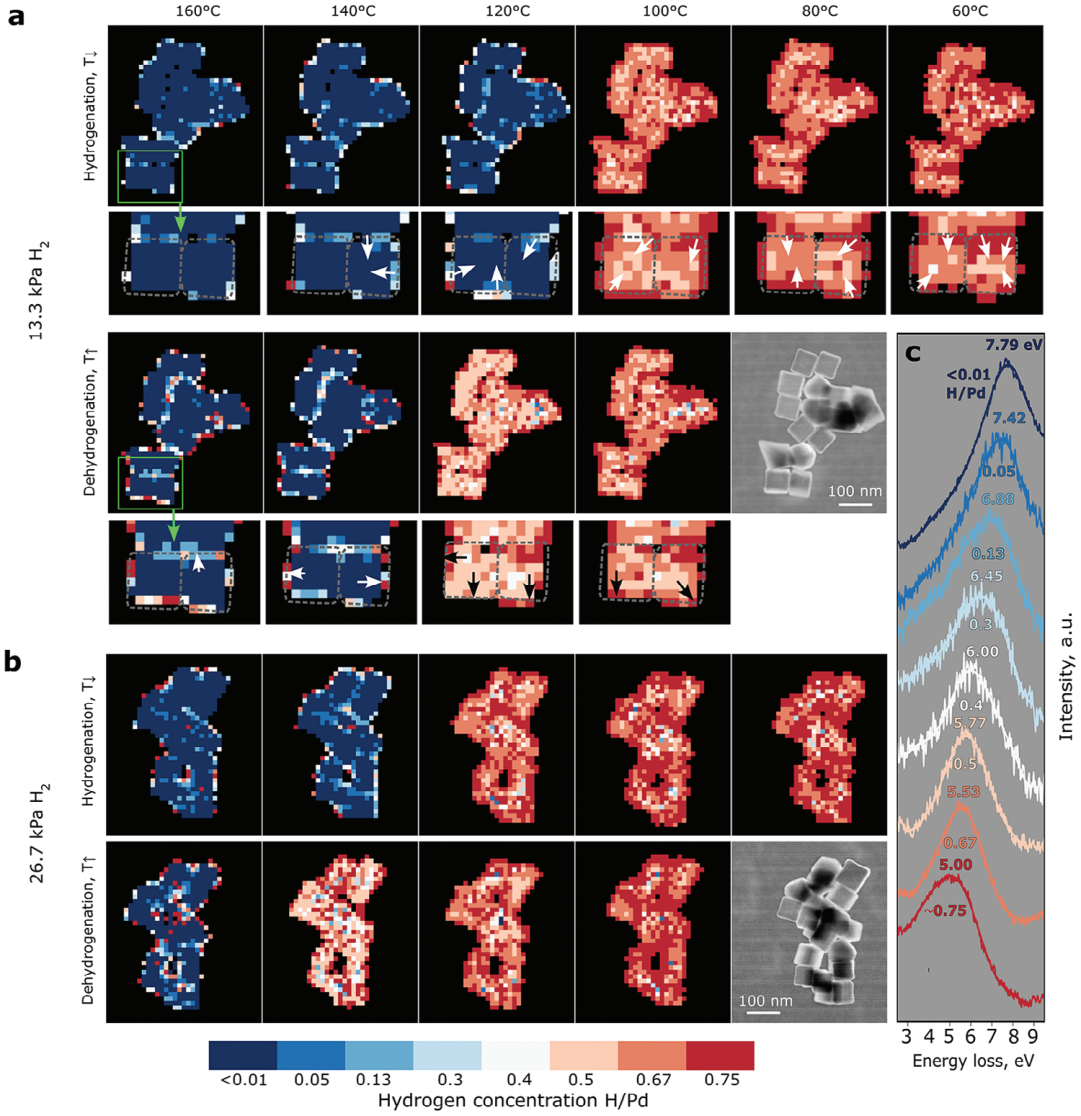
Measurement of the local hydrogen concentration. a) Isobaric series of 67 nm cubic Pd NPs during hydrogenation measured at constant hydrogen pressure *p*  =  13.3 kPa and varying temperature. Hydrogen absorption and hydride formation presented in the upper row appear from left to right with the temperature decrease. In the lower row, hydrogen release and hydride decomposition appear from right to left with the temperature increase. Magnified concentration maps of the area marked with a green rectangle are presented below each main map. Arrows indicate the direction of hydrogen concentration change in the magnified maps. b) Isobaric series of 67 nm cubic Pd NPs hydrogenation measured at constant pressure *p*  =  26.7 kPa. Hydrogenation presented in the upper row from left to right proceeds with the decrease of temperature. In the lower row, dehydrogenation from right to left appears with the increase in temperature. c) Experimental net EELS spectra on Pd NPs with different hydrogen concentrations after SiN background subtraction, fitted with a polynomial function for all concentrations.

Isobaric hydrogen concentration measurement series for PdH_x_ NPs at different H/Pd ratios are presented in **Figure** **3**c. We change the H/Pd ratio by varying the temperature while keeping the pressure constant. The experimental path is roughly shown in Figure 1 by the dotted black horizontal line representing the phases present in the PdH_x_ system at constant hydrogen pressure and different temperatures. Each column in Figure 3a,b corresponds to the same temperature to compare the hydrogen concentration in the NPs during hydrogenation and dehydrogenation cycles.

We determine the maximum peak position of the Pd bulk plasmon for every pixel in the EELS maps with a procedure described in the Supporting Information. Then the hydrogen concentration is prescribed to each plasmon position with respect to the simulations in Figure 2.

At 160 °C, for both investigated pressures the hydrogen concentration is found to be small in the NP, and the local concentration is determined to be < 0.01 H/Pd. This matches the phase diagram of Pd‐H with a solid solution limit of ≈0.047 H/Pd for 160 °C.^[^ ^40^ ^]^

### 4.1 Local Changes in Hydrogen Concentration During Hydrogenation/Dehydrogenation

During temperature reduction, the surface regions of ≈4 nm start to hydrogenate at higher temperatures than the bulk regions of the NPs. The thickness of the surface regions is equal to or below the resolution limit of ≈4 nm for the delocalized low‐loss EELS signal (see Supporting Information). With the decrease in temperature, the concentration at the surface grows. The inner parts of the NPs thereby stay in the α‐phase. This is evident comparing the maps at 160 and 120 °C at 13.3 kPa and the maps at 160 and 140 °C at 26.7 kPa in Figure 3. For example, at 13.3 kPa most surface regions have concentrations of 0.13 H/Pd at 160 °C. At 120 °C the average concentration increases to ≈0.40 H/Pd in the surface region. At 26.7 kPa and 160 °C, the initial surface concentration reaches 0.75 H/Pd in some areas. At a lower temperature of 140 °C the hydride starts to form at the whole surface including the places where NPs touch. The width of this outer region with gradually increasing concentration is ≈10–15 nm. In the enlarged region presented in Figure 3 below every map, the local changes in hydrogen concentration at 13.3 kPa are highlighted.

With further decrease in temperature, the NPs transform to the α΄‐hydride phase. There, the hydrogen concentration varies locally inside the NPs. This effect is even stronger than in the α‐phase. We consider this inhomogeneous distribution to be significant. Generally, the hydrogen distribution is similar to the previous case, with the core being in the α‐phase and higher concentrations in the outer regions, see Figure 3. At 100 °C and a pressure of 13.3 kPa, the NPs right after the phase change mainly show regions with 0.67 H/Pd concentration in the core. The shell has a higher concentration of 0.75 H/Pd. With a continuing decrease in temperature, the hydrogen concentration again starts to increase from the surface to the middle of the NPs, resulting in the growth of areas with 0.75 H/Pd concentration and a width of ≈10–20 nm. While at 100 °C there are also local regions with 0.50 H/Pd present, these regions are significantly depleted at 60 °C at the expense of regions with higher concentrations. At the higher pressure of 26.7 kPa, the rise of the concentration is even more prominent: areas with 0.75 H/Pd have significantly grown at 80 °C compared to the dataset at 120 °C, where 0.67 H/Pd concentration regions prevail. One can clearly see that the phase transformation and further hydrogenation of the Pd NPs start at the surface and proceed in the direction of the inner part of the NPs. This observation contradicts previous work,^[^ ^32^ ^]^ which concluded from pure thermodynamic arguments that the hydrogen concentration in surface and subsurface regions stays constant at intermediate values between those of α and α΄ phase.

A hysteresis is observed for all the isobaric series presented in Figure 3. For both constant pressure series of 13.3 and 26.7 kPa, the temperatures of phase change from α to α΄ during hydrogen absorption of the NPs are ≈20 °C lower than the temperatures for the reversed‐phase transition α΄ → α during hydrogen desorption. For example, during absorption at 13.3 kPa, NPs are in the α‐phase at 120 °C. During overall hydrogen release at the same pressure, the NPs are still in the hydride phase at 120 °C. A similar situation is observed at the higher pressure of 26.7 kPa during hydrogen absorption and desorption.

**Figure 4:**
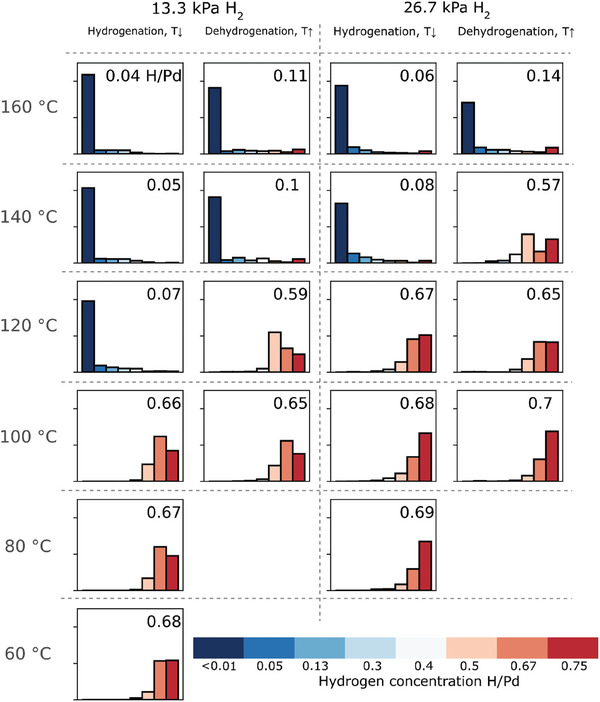
Histogram of the concentration distribution in the NPs extracted from the maps in Figure 3 representing the concentration maps to more generally observe the processes of hydride formation and decomposition. The number of pixels that belong to the same concentration was calculated for every map in Figure 3a,b, including the surface regions. Each graph displays the concentration distribution per number of pixels for every map. The vertical axis in each graph represents the number of pixels in a range from 0 to 500. The histograms show the hydrogen distribution during hydrogenation of the NPs carried out at 13.3 kPa H_2_ in a) and b), extracted from Figure 3a, and carried out at 26.7 kPa H_2_ in c), extracted from Figure 3b.d) The histograms represent the hydrogen distribution during dehydrogenation carried out at 26.7 kPa H_2_, extracted from Figure 3b. The number in the upper right corner of each diagram gives the average hydrogen concentration in the NPs, calculated by averaging over all NPs in the field of view.

During dehydrogenation, hydrogen first leaves the middle of the NPs, according to Figure 4. With the increase of temperature from 100 to 120 °C, the hydrogen concentration starts to reduce in the core of the NPs going from 0.67 to 0.5 H/Pd.

When the NPs core parts turn back to the α‐phase at 140 °C (13.3. kPa) and at 160 °C (26.7 kPa) during dehydrogenation, the major part of the surface regions remains in the hydride phase (Figure 3). Local concentrations of ≈0.5–0.75 H/Pd are still detected at the surface. The remaining hydride phase concentrations at the surface are observed 5 min after the dehydrogenation of the center of the NPs.

### 4.2 Statistical Representation of Hydrogen Concentration Changes During Hydrogenation/Dehydrogenation

Another way to represent the change in local hydrogen concentration during the hydrogenation/dehydrogenation processes is depicted in **Figure** **4**. Each histogram in this figure, extracted from the specific maps in Figure 3a,b, shows the distribution of hydrogen concentration in the NPs at a given temperature. The hydrogen distribution is calculated by counting the number of pixels with the same concentration in each map. Consequently, the height of each bar in a histogram displays the number of pixels corresponding to a specific concentration. This gives a statistical representation of the changes in the hydrogen concentrations in the NPs during hydrogenation/dehydrogenation. For example, one can see that the bars representing areas with 0.13–0.40 H/Pd concentration increase during the early stages of hydride phase formation, when the core of the NPs is still in the α‐phase for both pressures 13.3. and 26.7 kPa in Figure 4. We attribute this observation to the onset of hydride formation in the near‐surface region of the NPs, which can also be seen in Figure 3a,b. With temperature reduction from 160 to 140 °C at 26.7 kPa, the intermediate concentrations of 0.13 and 0.3 H/Pd also increase at the surface. The averaged spectra corresponding to intermediate concentrations of 0.13 and 0.3 H/Pd are presented in Figure 3c. An enhanced content of hydrogen in the subsurface and beyond supports the theory of Sachs et al.^[^ ^32^ ^]^ for Pd clusters, suggesting that hydrogen can occupy the subsurface sites of Pd NPs with concentrations between α and α΄. In contrast to this model, in the current experiment the hydrogen concentration keeps increasing in the near‐surface region and reaches values of the hydride phase, see Figure 3. The histograms also help to visualize changes in local concentrations after the phase transition is completed. Initially, high bars for 0.50 and 0.67 H/Pd decrease when the temperature declines from 100 to 60 °C for 13.3 kPa pressure (Figure 4). Simultaneously, the bars for 0.75 H/Pd rise with the decrease in temperature. The same trends are visible for the hydrogenation at a higher pressure of 26.7 kPa. Moreover, here we can also clearly see the bars for a high concentration of 0.75 H/Pd.

During dehydrogenation, the numbers of regions with higher concentrations gradually decrease for both investigated pressures. The differences in the distribution of 0.50 and 0.67 H/Pd areas during hydrogenation and dehydrogenation become even more obvious when looking at the histograms. Right after full hydride formation during hydrogenation, 0.67 H/Pd areas prevail over 0.50 H/Pd areas at 100 and 120 °C, for both pressures. However, just before the phase transition from the hydride phase to the α‐phase during dehydrogenation, regions with 0.50 H/Pd become dominant at 120 °C and 13.3 kPa H₂, or at 140 °C and 26.7 kPa H₂, indicating hysteresis.

## 5 Change in Strain within the NPs During Hydrogenation and Dehydrogenation

**Figure 5:**
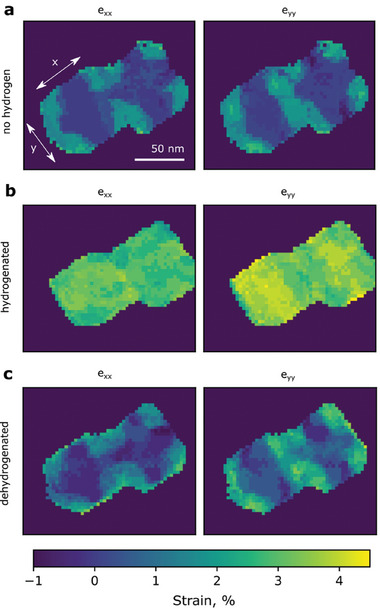
Elastic strain was measured with nanobeam electron diffraction in two adjacent 67 nm Pd NPs on SiN at different temperatures. e_xx_ and e_yy_ represent the elastic strain in lateral x and y directions, correspondingly. The reference strain state of the NPs is taken from metallic, unstrained Pd NP measured on lacey carbon. a) Strain maps of Pd NPs in metallic state acquired without hydrogen at 200 °C. The Pd crystal lattice is dilated at the corners of the NPs by ≈1.5%. b) Strain maps of fully hydrogenated Pd hydride NPs acquired at 13.3 kPa and 100 °C. After hydrogen absorption, the lattice of the NPs is overall dilated by ≈3–4% compared to the metallic Pd. c) Strain maps of dehydrogenated Pd NPs in solid solution state acquired at 13.3 kPa and 140 °C. After dehydrogenation, the strain distribution in the Pd NPs goes back to the configuration a) with dilation at the corners and more compressed regions in the middle of the NPs.

As discussed in the introduction, preexisting strains in nanosystems can modify hydrogen absorption/desorption and hydride formation processes.^[^ ^33^ ^]^ Inhomogeneous local strains are observed in the NPs in this study, as revealed in **Figure** **5**. According to the strain map of the NPs without hydrogen (upper row), the edge regions exhibit expansion (tensile strain) relative to the inner core sites.

The local strain in the NPs is measured in the presence and absence of hydrogen using the same atmosphere holder as for the STEM‐EELS experiments. We separate the effects of hydrogen absorption in the lattice from other possible causes of strain by locally evaluating the strain distribution in the absence of hydrogen on different substrates, as detailed in the Supporting Information. The strain values represent the lattice expansion or compression compared to the ideal case of the unstrained Pd lattice with 0% strain. The results are presented in Figure 5a–c. Before introducing hydrogen into the system, metallic Pd NPs show a strain distribution with dilation at the corners of ≈1.5%, at 200 °C. Temperature‐related changes of the lattice strain are small in the experimental temperature range and neglected in the calculation. Figure 5b represents the local strain in the same but fully hydrogenated Pd NPs at 100 °C and ≈13.3 kPa H_2_ pressure. The hydrogen‐containing NPs show an overall dilation of 3–4% compared to the metallic unstrained Pd lattice. This resembles the hydrogen‐induced expansion of free bulk PdH_x_ after hydride formation. The strain difference between the outer and inner parts of the NPs in the hydride phase is less pronounced than in the case of α‐phase NPs. However, the corners of the hydrogenated NPs (Figure 5b) are strongly dilated by ≈1% absolute strain as compared to the corners of NPs in the metallic state (Figure 5a). After a temperature increase to 140 °C, the NPs dehydrogenate, as shown in Figure 3. Then, tensile strain of ≈1.5–2.5% is observed at the corners (Figure 5c), similar to the first measurement without hydrogen.

## 6 Discussion

### 6.1 Hydrogenation/Dehydrogenation Process

The mean hydrogen concentrations detected in the Pd NPs overall align closely with the values expected for bulk. At higher temperatures, the solubility limit of hydrogen in the solid solution PdH_x_ α‐phase increases. For example, in bulk Pd, the maximum hydrogen concentration in the α‐phase reaches 0.03 H/Pd at 120 °C and 0.047 H/Pd at 160 °C.^[^ ^49^ ^]^ However, at 160 °C and 13.3 kPa, the observed hydrogen concentration in the surface region of the NPs (Figure 3) amounts to values of about x = 0.13 – 0.40 H/Pd, while the core remains in α‐phase. This proves the hypothesis of Sachs et al.^[^ ^32^ ^]^ that intermediate concentrations outside of the stability regions can exist at the surface of the NPs. At a higher pressure of 26.7 kPa, the concentration in the surface region of the NPs can reach values as high as 0.75 H/Pd while the interior of the NPs remains in the α‐phase. This increase in concentration and the formation of Pd hydride close to the surface goes beyond the assumption of Sachs et al.

Right after the phase transition, regions with 0.50 ± 0.05 and 0.67 ± 0.05 H/Pd are detected in the interior of the NPs. This value agrees with the minimum hydrogen concentration of 0.54 H/Pd in the bulk Pd hydride phase at 120 °C. During ongoing hydride formation with decreasing temperature, the areal density of regions with 0.50 H/Pd decreases, while at both pressures the density of regions with concentration of 0.75 H/Pd increases. The regions with enhanced surface concentrations extend to the interior of the NPs by as much as 10–30 nm (Figure 3). This increase in hydrogen concentration near the surface indicates that the hydrogenation of the NPs initiates at the surface and progresses inward. This trend is observed even when the NPs have entirely transformed to the hydride phase. During dehydrogenation, the minimum hydrogen concentration in the α΄‐phase is lower than during hydrogenation, with the majority of regions at 0.50 ± 0.05 H/Pd, see Figures 3 and 4. This suggests that the hydride phase reaches a meta‐stable condition at enhanced temperature with correspondingly lowered hydrogen concentration. This might result from an energy barrier that has to be surpassed for precipitation of the α‐phase during dehydrogenation. The energy barrier can be related to the coherency stresses^[^ ^28^, ^57^, ^58^, ^59^ ^]^ or to the conventional nucleation barrier for interface formation. This effect has to be clarified by studying hydrogenation and dehydrogenation in isothermal conditions.

### 6.2 Strain Impact on Hydrogenation/Dehydrogenation

**Figure 6:**
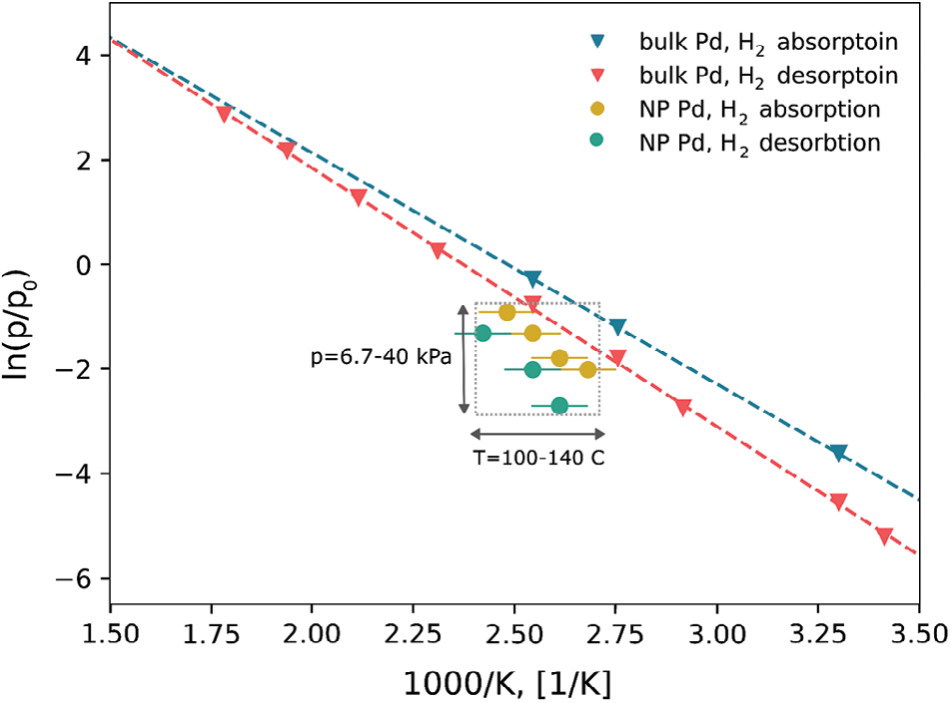
Van't Hoff plot for 67 nm Pd NPs and bulk Pd.^[^ ^49^ ^]^ The plateau pressure and temperature values for the phase transitions during hydrogenation/dehydrogenation of the NPs were concluded from the data in Figure 4 and additional experiments. For instance, the phase shift from α‐ to α΄‐phase happens between 120 and 100 °C at 13.3 kPa during hydrogenation. Then the plateau pressure and temperature are estimated as 13.3 kPa and 110 °C, respectively.

The lattice of defect‐free bulk Pd expands isotropically during hydrogen absorption if no other factors are involved. If the metal lattice is strained anisotropically by elastic constraint conditions such as particle adhesion to a rigid substrate or by the presence of edges and corners,^[^ ^33^ ^]^ the local elastic strain distribution can modify the hydrogen distribution in the metal due to the Gorsky effect.^[^ ^60^ ^]^ In our experiment, the in situ hydrogenation and dehydrogenation start at lower pressures and higher temperatures for NPs compared to bulk PdH_x_. **Figure** **6** summarizes the measured plateau pressures for the phase transition of the NPs core and bulk, both in absorption and desorption. The 67 nm NPs show deviations from bulk behavior, as revealed in this work. This may be attributed to the boundary conditions and the resulting inhomogeneous strain state observed in the NPs (see below), as well as to differences between the surface region and the NP core.

As shown in Figure 5, NPs are initially inhomogeneously strained at 200 °C in the absence of hydrogen. Regions at the corners of the NPs, extending inward by ≈15 nm, exhibit a tensile strain of ≈1–2% compared to the centers. These strain fields may modify the local hydrogen concentration, increasing it in strained regions and decreasing it in compressed regions. The extension of the initial strain field explains the formation of a higher hydrogen concentration region of about the same size.

This effect can be qualitatively discussed according to the local elastic stress impact on the chemical potential of hydrogen in the metal lattice. The chemical potential of hydrogen with stress contributions (which can be linked to the strain by the stiffness tensor $\sigma \;=C(\varepsilon -\varepsilon_{0})$) is given by:^[^ ^24^, ^33^ ^]^

(1) $$\mu_{H}=kTln\frac{c_{H}}{1-c_{H}}+\varepsilon_{0}+\varepsilon_{HH}zc_{H}-v_{0}\eta_{H}tr\left(\sigma \right)\;$$

Here, ɛ_0_ is the site energy of hydrogen atoms on interstitial sites, ɛ_*HH*_ is the attractive H–H pair interaction energy parameter, *z* is the number of next neighbors of interstitial sites, *v*_0_ is the partial molar volume of interstitial sites, η_*H*_ is the hydrogen‐induced dilatation coefficient of the Pd metal lattice, ***ɛ*** is the strain of an NP, ***ɛ***_0_ is the pure H‐induced strain, and *tr*(***σ***) is the resulting sum of axial stresses in the system. Without hydrogen ***ɛ***_0_ = 0. Tensile strain in the corners, see Figure 5a, results in ***ɛ*** > 0 and $\sigma \;=C(\varepsilon -\varepsilon_{0})$> 0. Assuming a constant chemical potential in the NPs, this requires a higher hydrogen concentration in the corner positions. This meets our experimental observation.

In thermodynamic equilibrium, the chemical potential µ_*H*_ is constant throughout the system. Consequently, local stresses and the associated local strains must be balanced by variations in local hydrogen concentration: lattice dilation at the corners of the NPs in the α‐phase can reduce the potential barrier, facilitating the onset of hydrogenation at lower pressures and higher temperatures. The same reasoning can be applied to the NPs in the α΄‐phase. There, the inhomogeneous distribution of hydrogen in the hydrogenated NPs presumably is also caused by the preexisting strain in the lattice. However, the average strain ***ɛ*** in the PdH_x_ NPs in the α΄‐phase, as well as the strain in the inner parts of the NPs in the α‐phase, remains close to the H‐related strain ***ɛ***_0_ of bulk PdH_x_. Thus, the van't Hoff lines of the PdH_x_ NPs are only slightly shifted with respect to the PdH_x_ bulk lines.

## 7 Summary

In this work, we establish a new approach to measure local hydrogen concentrations in Pd NPs with a minimum spatial resolution of 3–4 nm, by using the EELS plasmon maximum peak position. Gradual shifts of the Pd bulk plasmon with respect to the hydrogen concentration, shown in theoretical calculations,^[^ ^53^ ^]^ are used to experimentally determine the local hydrogen content in NPs. Surface plasmons affect the absolute plasmon position only for the outer 4 nm surface region of the NPs and need to be considered in the data treatment. The method offers a way to observe the hydrogenation and dehydrogenation processes from the early stages of hydrogenation while the NPs stay in the solid solution phase, to the later stages when the NPs are fully transformed to the hydride phase, in great detail at the nanoscale.

During the early stages of hydrogenation, hydrogen starts to enter the NP from the surface. We observe hydrogen concentrations from 0.13 to 0.4 H/Pd in the near‐surface region of the NPs, originating from a higher hydrogen uptake in surface and subsurface sites,^[^ ^32^ ^]^ but also from the initial tensile strain conditions. Pd hydride starts to form at the surface of the NPs with concentrations from 0.3 H/Pd. Remarkably, after the phase change of the entire NPs, the concentration increase still continues in the surface region, clearly marked by the increase up to 0.75 H/Pd regions at the surface in the sequence of increasing hydrogen concentrations in fully hydrided NPs. The concentration increase at the surface regions goes beyond the previous models of Sachs et al., who assumed surface and subsurface regions to stay at medium concentrations between α and α΄, with the absence of hydride formation in these regions.

The proposed method opens various opportunities to investigate the local hydrogen concentration in metals with nanometer resolution in wide temperature ranges between 25 and 1000 °C and pressures up to 1–2 bar, which is important in the fields of hydrogen storage, sensing, and embrittlement. The method could be of particular interest for studying hydrogen storage mechanisms at the nanoscale in Mg, Al‐containing alloys, and other related materials. The effect of hydrogenation on the shift of the bulk plasmon in these materials should be estimated by optical measurements or/and simulations of the dielectric functions with various concentrations of hydrogen in the material, beforehand. The versatility of in situ TEM also allows to link together local strain, microstructure, and hydrogen concentration, opening up a new chapter in hydrogen research.

## Conflict of Interest

The authors declare no conflict of interest.

## Author Contributions

S.K., S.W., and A.P. performed conceptualization. S.K., S.W., D.R., P.V., and A.P. performed the methodology. S.K. performed an investigation. S.K., S.W., and A.P. visualization. D.R. and P.V. performed recourses (sample synthesis). A.P. performed project administration. S.W., A.P., and P.V. performed supervision. S.K. wrote the original draft. S.K., S.W., D.R., P.V., and A.P. wrote, review and edited the final manuscript.

## Supporting information



Supporting Information

## Data Availability

The data that support the findings of this study are available from the corresponding author upon reasonable request.
