# Supplementary material for: Local Hydrogen Concentration and Distribution in Pd Nanoparticles: An In Situ STEM‐EELS Approach
Source: Small. 2024 Dec 8;21(16):2407092. doi: 10.1002/smll.202407092 (PMC12019903; doi:10.1002/smll.202407092)
Supplement: Supplementary file 1 — Supporting Information [file SMLL-21-2407092-s001.pdf]

NANO · MICRO
small

Supporting Information

for *Small*, DOI 10.1002/smll.202407092

Local Hydrogen Concentration and Distribution in Pd Nanoparticles: An In Situ STEM-EELS Approach

Svetlana Korneychuk, Stefan Wagner, Darius Rohleder, Philipp Vana and Astrid Pundt*

Determining local hydrogen concentration and distribution in Pd nanoparticles: An *in-situ* STEM-EELS approach

– Supporting information –

S. Korneychuk [1,2*], S. Wagner [1], D. Rohleder [3], P. Vana [3,4], A. Pundt [1]

[1] Institute for Applied Materials – Materials Science and Engineering (IAM-WK), Karlsruhe Institute of Technology, Engelbert-Arnold-Straße 4, 76131 Karlsruhe, Germany

[2] Karlsruhe Nano Micro Facility (KNMF[i]), Karlsruhe Institute of Technology, Herrmann-von-Helmholtz-Platz 1, 76344 Eggenstein-Leopoldshafen, Germany

[3] Institute of Physical Chemistry, Georg-August-University Göttingen, Tammannstr. 6, 37077 Göttingen, Germany

[4] Wöhler Research Institute for Sustainable Chemistry (WISCh), Georg-August-University Göttingen, Tammannstr. 2, 37077 Göttingen, Germany

*Corresponding author. Email: svetlana.korneychuk@kit.edu

ORCID ID Svetlana Korneychuk: 0009-0007-1231-2799

1. Overview of other methods for hydrogen detection

Standard methods of observing hydrogen absorption and desorption, for instance, gas gravimetry or gas volumetry, provide only spatially averaged information. Cutting-edge atom-position resolving methods like atom probe tomography APT [1] allow for one static analysis per sample as the method is destructive. On top of this, APT requires successful atom position freezing, which is challenging, if not impossible, in the case of hydrogen. Further, secondary ion mass spectroscopy (SIMS) [2] or proton-proton microscopy [3,4] detect hydrogen in materials locally. While SIMS has a lateral resolution in the 100 nm range and nm resolution in the vertical direction, it is very sensitive but not quantitative. Proton-proton microscopy is quantitative but has a resolution of about 1 μm in lateral directions with a depth resolution down to 5 μm. All three methods do not provide real-time information. We demonstrate here that the combination of transmission electron microscopy (TEM) with *in-situ* gas loading can yield information on the nanometer scale and even atomic level while observing the hydrogen interaction with the sample in real time.

Hydrogen can directly be detected with EELS by measuring the H K-edge signal. However, the cross-section of an H atom is very low, the hydrogen K-edge lies in the low loss region where its detection is hindered by the tail of the zero-loss peak, plasmon excitations from Pd and material of the substrate. Finally, the hydrogen gas surrounding the sample in our experiment gives the main contribution to the H signal.

Hence, in our work we determine the Pd bulk plasmon position as a function of the hydrogen concentration.

2. Description of cubic Pd NPs sample preparation

As the width of the two-phase region relates to the hydrogen storage capacity of a metal-hydrogen system, a large width is commonly beneficial for storage applications. Increasing the NP size will increase the storage capacity of the NP by increasing the number of inner bulk-like sites. Thus, a particle size of 67 nm is chosen in this study. A cubic particle shape is beneficial to achieve a maximum NP packing density.

Cubic palladium NPs of 67 ± 7 nm size were synthesized in two stages. Firstly, 18 ± 2 nm cubic NPs were produced via a seedless method by reducing H_2PdCl_4 in ascorbic acid in aqueous solution in the presence of cetyltrimethylammoniumbromide (CTAB) as surfactant [5,6]. Secondly, these cubic particles served as seeds to obtain Pd cubes with larger size by controlling the seed- and feed solution. The surface of the cubic Pd NPs was subsequently functionalized with a poly(N-isopropylacrylamide) (pNIPAM, M_n = 26 kg mol^{-1}, *Đ* = 1.07) from controlled reversible addition-fragmentation chain-transfer (RAFT) polymerization via a grafting-to approach to ensure the nanocube stability. Pd nanoparticles investigated with STEM EELS are covered with this polymer shell. For the TEM analysis, the Pd NPs are drop casted in ethanol solution on the SiN window of the chip.

3. Simulations of the PdH_x plasmon shift

In the simplest case, the imaginary part of the dielectric function determines the EELS signal in the low loss region if no retardation losses are involved [7]. However, the plasmonic response of the nanoparticle can be influenced by its shape and size, the exact place of the local EELS measurement, as well as the substrate and the medium around the particle. Hence, to elucidate possible changes of the EELS signal from the ideal case, we carried out EELS simulations on PdH_x 67 nm nanoparticles with the MNPBTEM toolbox [8] using the simulated dielectric functions from Silkin *et al.* [9] (Figure 2 of the main text). The refractive index of the surrounding medium is chosen to be 1.5 to account for the presence of the NIPAM polymer, gas medium, and possible carbon contamination caused by the electron beam. The energy shift of the PdH_x NP plasmon is almost linear with the increase of hydrogen concentration x, as shown in Figure 2a of the main text by the gray circles. We approximate these simulated values with the linear function $E_{bulk}(eV) = 7.80 - 3.64x_{H/Pd}$ (1). This approximation can be used to estimate the H concentration in Pd with STEM EELS in the samples with thickness above 40 nm excluding edges and surfaces. For a more sophisticated approximation of the plasmon shift with respect to the hydrogen concentration, additional experimental measurements and/or simulations of the PdH_x system are required.

Simulated EELS spectra acquired in the middle of the 66 nm $PdH_{0\leq x\leq 1}$ cubic particle with uniform concentration (Figure 2e from the main text, spectra plotted with solid lines) are very similar to the imaginary part of the input dielectric function from the work of Silkin [9]. This also accounts for all local positions in the particle that are more than 4 nm away from the particle surface.

At the local position of the edge and surface of the NPs, a red shift of the plasmon maximum is detected in the simulations (Figure 2a, e of the main text), with similar dependency on the hydrogen concentration. This shift is presented on the Figure 2a in the main text for two positions – at the very edge of the particle illustrated by green triangles and right outside of the particle shown by purple crosses. The latter is only sensitive to the surface modes and, therefore, shifted to the lowest values. In the STEM EELS experiment, due to the delocalization of the low loss EELS signal of about 4 nm [7], signals from both edge and surface positions will be measured. Therefore, this shift is considered for the accurate interpretation of the hydrogen concentration at the edges of the particle. The red shift of about 1 eV from 7.8 eV to 6.9 eV is the largest in case of pure metallic NPs (Figure 2a, main text for $x = 0\ H/Pd,$). For pure PdH_x dielectric particles, the calculated shift at the surface region is much lower, going from about 0.5 eV for $x = 0.125\ H/Pd$ to about 0.2 eV for $x \geq 0.3\ H/Pd$ (Figure 2a, main text for $= 0.3 - 1\ H/Pd$). The simulations show that the hydride formation at the edge of the particle with core concentration below 0.01 H/Pd can only be distinguished from red shift at the surface of the pure particle with the same concentration, if the maximum peak plasmon position at the edge of the particle is lower than 6.75 eV (maximum peak position of red shift nearby the surface 6.9 eV – measurement error of 0.15 eV).

Shell thickness. In EELS, electrons with a high kinetic energy pass by or penetrate through a metallic nanoparticle, excite particle plasmons, and lose part of their kinetic energy. The shift of the Pd plasmon is simulated with the MNPBTEM toolbox [8] which employs the boundary element method [10] for simulation of EELS signals. In this method, the boundaries between different dielectric media, in our case, the boundaries between the core and shell of NPs and vacuum, are discretized by boundary elements. This approach assumes that an isotropic dielectric environment is separated by abrupt interfaces or surfaces. Artificial surface charges and currents are attached to these surface elements. Given these conditions, Maxwell's equations are solved to determine the energy loss of the electron of the primary beam as it penetrates through the model particle exciting plasmonic response. By monitoring this energy loss as a function of electron beam position, a detailed map of the localized plasmonic fields can be obtained. To accurately compute the dielectric response in the middle of the PdH_x shell, the surface that determines the boundary between the shell and vacuum should have at least three more surface elements than the inner surface, which indicates the boundary between the core and the shell. Therefore, for a thinner shell, more surface elements are required. However, decreasing the shell thickness to 1 nm, as it would resemble the thickness of the subsurface region of clusters, would lead to a surge in computational time, while it would not alter the results. Hence, a thickness of 3 nm for the shell was chosen as a compromise between computational time, spatial resolution of low loss EELS of about 4 nm and expected shell width.

3.1. Influence of the NP shape on the PdH_x plasmon shift

The EELS signal collected in the current experiment is averaged over the thickness of the NP in Z direction. Therefore, the shape and the size of the core-shell nanoparticle can influence the shift of the Pd plasmon. Large cubic core-shell NPs represent the simplest case where the material in Z direction consists mostly of the same phase for 66 nm NP with 1 nm or 3 nm shell (Supporting Figure S1b).

In the case of a spherical particle with the same diameter, the signal collected near the interface between the shell and core (marked with dot 2 in Supporting Figure S1) contains a significant contribution from the $PdH_{0.6}$ shell. To account for the possible influence of the NP shape, we carried out simulations with the MNPBTEM toolbox on cubic and spherical core-shell 66 nm NPs with a metallic core and $PdH_{0.6}$ shell with 1 nm or 3 nm thickness (Supporting Figure S1). The simulations show that the plasmon maximum of a cubic particle everywhere in the core does not depend on the presence of the 1 nm or 3 nm shell. On the other hand, the shape of the plasmon signal of a spherical NP in the core with close proximity to the shell significantly depends on the shell thickness (Supporting Figure S1a, marked with dot 2). In the hypothetical case of the 3 nm shell, the plasmon signal does not have a pronounced maximum peak anymore. In an experiment, this could potentially lead to a misinterpretation of the concentration value in the core close to the shell. When the shell is thinner, the influence of the shell is less significant. In conclusion, when this method is applied to smaller NPs or to NPs with shapes other than cubic, simulations should be conducted in advance to account for the possible influence of the NP shape and size on the plasmon shift, in addition to the hydrogen concentration change.

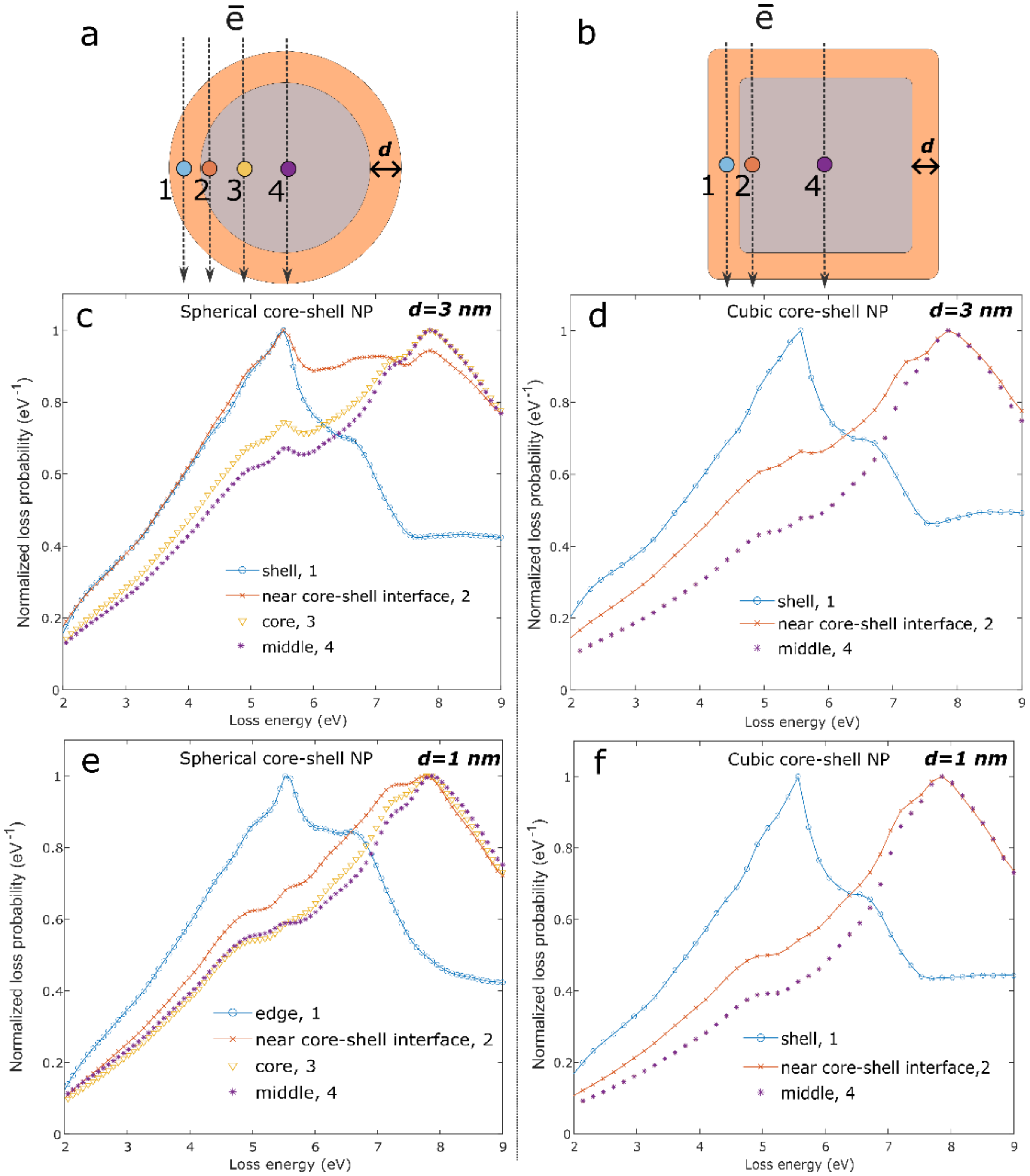

Supporting Figure S1. *Simulations of the plasmon signal from cubic and spherical 66 nm core-shell NP with a metallic core and* $PdH_{0.667}$ *shell conducted with the MNPBTEM toolbox* [8] *for shell thicknesses of 1 nm and 3 nm. a. Sketch of the spherical core-shell NP with a diameter of 66 nm and shell with a thickness d of 1 nm or 3 nm. Four points indicate different positions where EELS spectra are simulated. b. Sketch of the cubic core-shell NP with a size of 66 nm and shell with a thickness d of 1 nm or 3 nm. Three points, similar to a, indicate different positions where EELS spectra are simulated. Spectra simulated in 1-4 points in the spherical NP with a shell of 3 nm (c), 1 nm (e), and in the cubic NP with a shell of 3 nm (d) and 1 nm (f). Already at position 2, about 3 nm from the core-shell interface towards the middle of the NP, the contribution of the core signal is visible and dominates the plasmon signal.*

4. Measurements of PdH_x plasmon shift with TEM

We study hydrogen uptake and release in Pd NPs by *in-situ* TEM with an *in-situ* gas cell: Atmosphere AX from Protochips in a ThermoFisher Scientific Themis Z electron microscope. In this setup, the sample is tightly sealed between two MEMS chips and analyzed through electron-transparent SiN windows. The NPs are stable below 200 °C. To observe the formation of the palladium hydride phase in this temperature range, hydrogen pressures up to half atmosphere are utilized. Prior to the main experiment, Pd nanoparticles are purged with 0.5 bar of hydrogen for 5 minutes, which is pumped away afterwards. This procedure is repeated three times to remove a potential oxide layer from the surface of Pd nanoparticles [11]. Afterwards, the sample is purged with ultrapure Ar gas.

EELS measurements are carried out at the Themis Z equipped with Continuum Gatan spectrometer and CMOS detector. The formation of the Pd hydride phase is observed in real time with monochromated STEM-EELS. The influence of the SiN membranes on the path of the electron beam is minimized by choosing 80 keV acceleration voltage, which allows to significantly reduce the impact of Cherenkov radiation emission on the bulk plasmon signal. The energy resolution, measured through the membranes and estimated as full width at half maximum (FWHM) of the zero-loss peak, is 150 meV. The spatial resolution for detecting PdH_x at 80 keV can be estimated from the delocalization of the low loss EELS signal represented by the radius of the scattering probability in real space containing 50% of all inelastically scattered electrons: $L_{50} = 0.5\lambda/\theta_E^{3/4}$ where λ is the wavelength of the electron and $\theta_E \approx E/2E_0$ is the characteristic scattering angle [7]. This results in about 3-4 nm resolution for the losses of 7.7 eV and 5.5 eV. Due to the level of EELS signal noise and the finite energy resolution, we can determine the position of the plasmon peak with the best precision of about ±150 meV. This value is computed as a standard deviation of the measured maximum peak positions of the bulk Pd plasmon carried out in the atmosphere holder.

As the initial spectra also contain the contribution from the SiN membranes, it is removed beforehand, by subtracting the reference SiN signal acquired only on an amorphous SiN membrane. Due to variations of the background level in the EELS signal, this method is more efficient to determine the maximum bulk plasmon position rather than fitting it with gaussian functions. Due to the level of EELS signal noise and the finite energy resolution, we can determine the position of the plasmon peak with the best precision of about ±150 meV. Thus, local concentrations are discriminated with respect to this value with an error of ±0.05 H/Pd.

We determine the maximum peak position of the Pd bulk plasmon for every pixel in the EELS maps presented in Figure 3 in the main text. Individual spectra from every pixel are fitted with a polynomial, and then the local maximum of this function is calculated. The experimental spectra averaged over several maps at various temperatures, fitted with polynomial functions are presented in Figure 3c in the main text. The EELS energy range from 4 eV to 8 eV is divided into 8 regions which can be sufficiently separated with given energy resolution. Each region corresponds to a certain concentration extracted from the simulation in accordance to Figure 2 in the main text. The measurement error determines the minimum width of the regions. According to the simulations in Figure 2 in the main text, the hydrogen concentration at the surface region of about 4 nm is estimated using lower reference energy values of the plasmon peak positions. This correction is applied only to the surface regions of NPs which face the outside medium, not each other.

4.1. Stability of PdH_x under the electron beam

It was reported in the works of Baldi [12] and Narayan [13] that exposure to the electron beam triggered hydrogen release in the hydrogenated NPs. The heat transferred by the electron beam in the environmental TEM (E-TEM) was shifting the thermodynamic equilibrium of the Pd-H system to the solid solution phase. However, no accidental release of hydrogen is detected in our study upon acquisition of the EELS maps. For example, an exposure to the electron beam does not lead to dehydrogenation of the PdH_x NPs, as shown in Supporting Figure S2 by two consequent EELS maps acquired first at a 100 °C and then at 110 °C, both at the same pressure of 13.3 kPa H_2. This can be attributed to several factors: in the single phase regions our measurement is carried out at temperatures and pressures that are far away from the phase transition conditions so that the heating by a couple of degrees does not shift the system equilibrium to the other phase; only during directed phase transitions by changing the experiment temperature in the chamber (see below) local heating by the electron beam might have a small impact on the thermodynamic stability of the phases. Additionally, the environment is more thermally stable inside the chip, acting as a heat bath compared to the large chamber of the E-TEM. Finally, the polymer shell of our NPs can play a role. In general, it is hard to estimate the exact amount of heat transferred by the electron beam. It depends on many factors, such as electron current, thermal conductivity of the nanoparticles and substrate, and the size of the particles or agglomerates. For instance, it has been shown [14] that the heat transfer to a thin film of α-Al_2O_3 varies from about 1 K for currents below 1 nA to about 20 K for currents of about 15 nA at 80 keV. In the current study, a current of 50-100 pA is used, leading to small expected T effects.

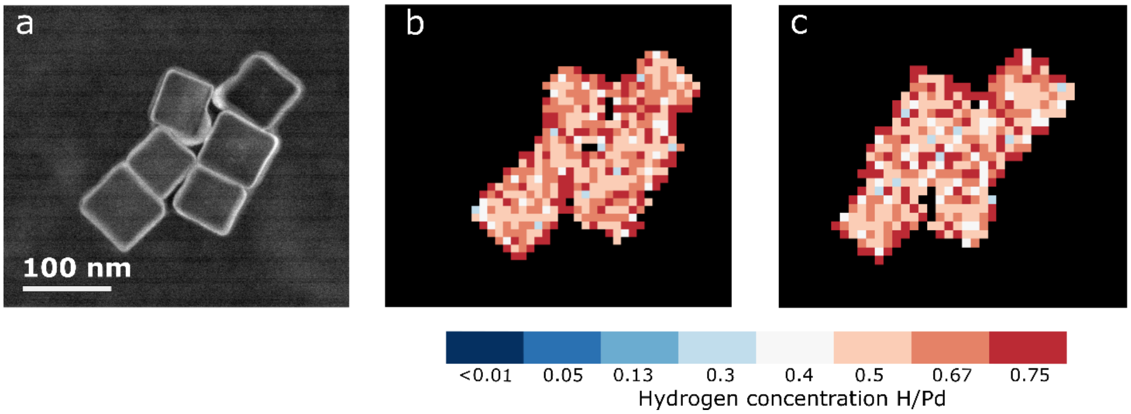

Supporting Figure S2. *Assessment of the electron beam influence on the dehydrogenation of Pd NPs. a. ADF-STEM image of 67 nm Pd NPs. b. Hydrogen concentration map acquired in area a. at 100 °C and 13.3 kPa H_2. c. Hydrogen concentration map acquired in area a. at 110 °C and 13.3 kPa H_2 after the map b.*

Minimizing Cherenkov radiation by using 80 keV

Cherenkov radiation occurs during TEM experiments when an electron of the primary beam travels through a dielectric medium (a TEM sample) with a speed greater than the speed of light in this medium [7]. This effect can usually be observed in materials with high refractive index, such as SiN at high acceleration voltages. This radiation creates an unwanted contribution to the low loss EELS signal which could compromise signal interpretation. One of the ways to minimize the impact from Cherenkov radiation is to lower the acceleration voltage of the microscope [15]. Cherenkov radiation caused by the presence of SiN

membranes was minimized by acquiring EELS spectra at 80 keV in the present study, as shown in Supporting Figure S3.

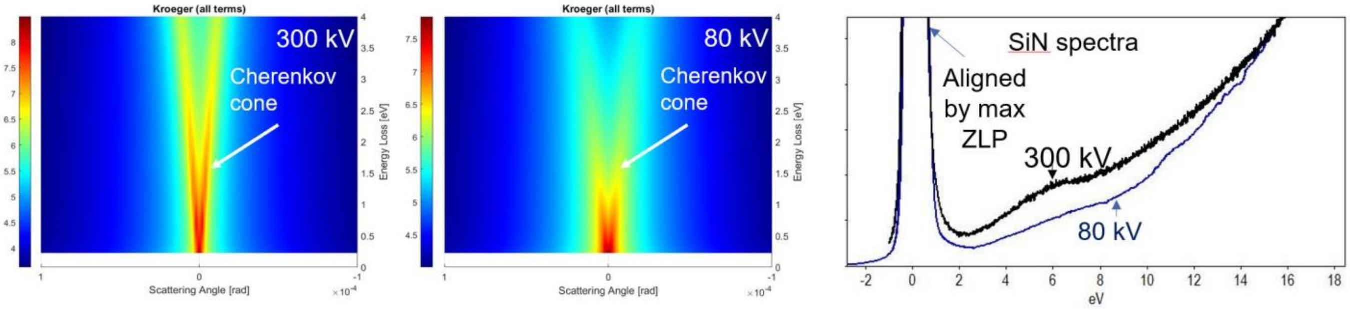

Supporting Figure S3. *Simulated Cherenkov radiation* [7] *at 300 keV and 80 keV for a SiN membrane. On the right – experimental spectra at 300 keV and 80 keV. Cherenkov radiation is strongly reduced at 80 keV.*

4.2. Shift of palladium bulk plasmon in vacuum

The Pd bulk plasmon shift was also measured in vacuum on lacey carbon substrate without hydrogen. The Pd plasmon at the surface of the particles is by about 1 eV lower than at the core of the particles, as illustrated in Supporting Figure S4.

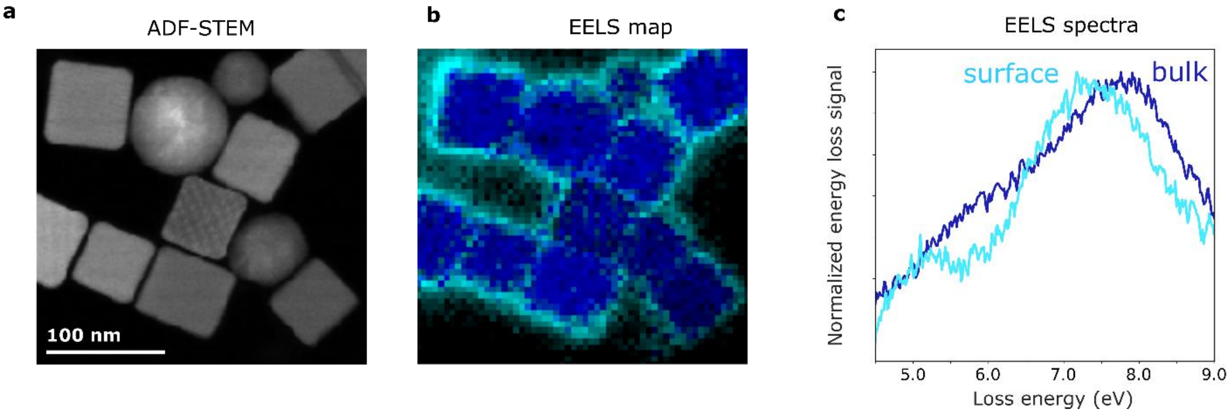

Supporting Figure S4. *a. ADF-STEM image of 67 nm cubic Pd nanoparticles. b. STEM-EELS map of Pd bulk plasmon at the surface (light blue) and in the core of the particles (deep blue). c. Corresponding EELS spectra for surface (light blue) and core (deep blue) of the particles.*

4.3. Concentration maps without binning

The initial EELS maps served for calculating the hydrogen concentration in Figure 3 in the main text are rebinned for a better signal to noise ratio with the final pixel size of 9.6 nm. For comparison, the unbinned concentration maps are presented in Supporting Figure S5 with a pixel size of 4.3 nm. These maps prove that the hydrogen concentration starts to grow at the surface regions with thickness of about 4 nm while the concentration in the core of the particles remains below 0.01 H/Pd.

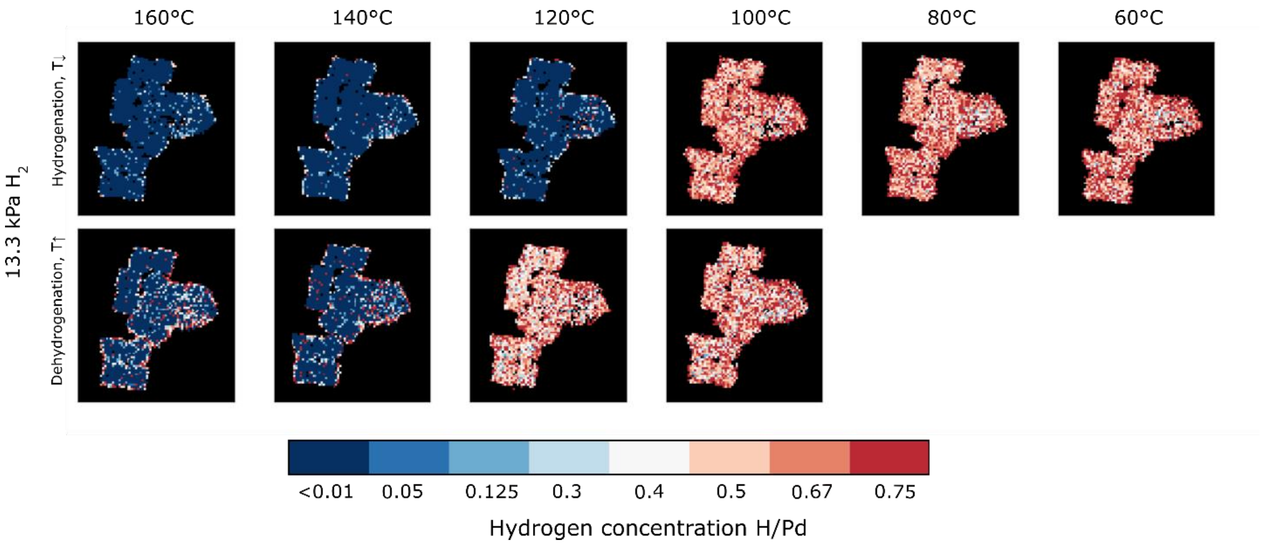

Supporting Figure S5. Local hydrogen concentration maps without binning from Figure 4 in the main article*. Isobaric series of 67 nm cubic Pd NPs during hydrogenation measured at constant pressure* $p = 13.3\ kPa$ *and varying temperature. Hydrogen absorption and hydride formation presented in the upper row appear from left to right with the decrease of temperature. In the lower row NP hydrogen release and hydride decomposition appear from right to left with the increase of the temperature.*

4.4. Hydrogen concentration at the surface before and after the complete hydrogenation

To eliminate a possible influence of the electron beam on the nanoparticles after multiple acquisitions, the hydrogen concentration was measured right before hydrogenation at 13.3 kPa and 140 °C (Supporting Figure S6a) and, excluding intermediate acquisitions, right after the dehydrogenation as well at 13.3 kPa and 140 °C (Supporting Figure S6b). A higher concentration of hydrogen at the surface after dehydrogenation was still detected during this measurement. This confirms that the surface of the nanoparticles stays hydrogenated in a vacuum after dehydrogenation of the core, at least for 5 minutes after dehydrogenation.

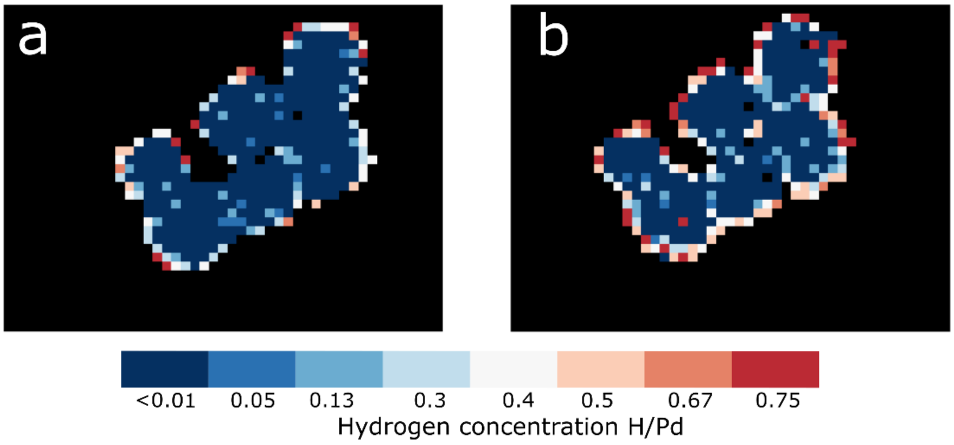

Supporting Figure S6. *a. Hydrogen concentration in the Pd 67 nm nanoparticles before hydrogenation of the core at 13.3 kPa and 140 °C. b. Hydrogen concentration measured in the same Pd 67 nm nanoparticles after dehydrogenation of the core at 13.3 kPa and 140 °C.*

5. Measurements of strain in PdH_x NPs with TEM

The strain distribution in the nanoparticles is detected via nanobeam electron diffraction using the Nanomegas precession system at 300 keV. The diffraction patterns are collected with a pixelated detector Dectris Quadro. In this setup, strain can be assessed locally with about 3 nm spatial resolution. The algorithm to measure strain is internally developed and based on the work of Béché *et al.* [16]. Peak finding procedure was implemented from the pixStem software [17].

5.1. Strain measurement on different substrates

We separate the effects of hydrogen intercalation in the lattice from other possible causes of strain by locally evaluating the strain distribution without hydrogen. Firstly, the strain is measured for particles on lacey carbon (Supporitng Figure S7) or amorphous SiN (Supporitng Figure S8) substrates and at different temperatures without the presence of hydrogen. At room temperature, the nanoparticles are less strained on both substrates than at higher temperatures, which is due to the differences in the thermal expansion between the NPs, polymer coating, and the substrates. With an increase in temperature, already at 80 °C, the strain at the corners of the NPs lying on the SiN substrate rises, reaching about 1.5% in dilation (Supporting Figure S9).

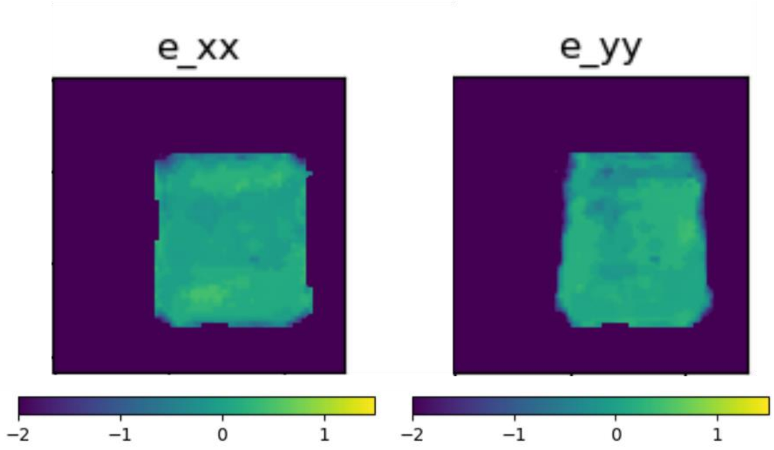

Supporting Figure S7. *Strain in 67 nm Pd nanoparticles measured at room temperature on lacy carbon in vacuum.*

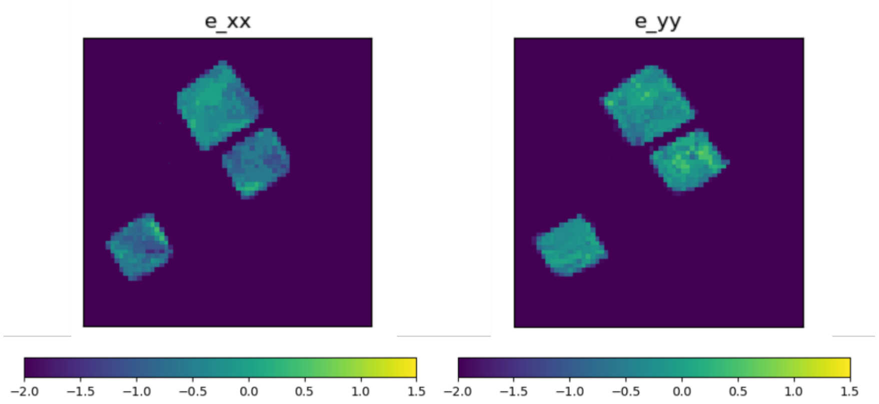

Supporitng Figure S8. *Strain in 67 nm Pd nanoparticles measured at room temperature on SiN in vacuum.*

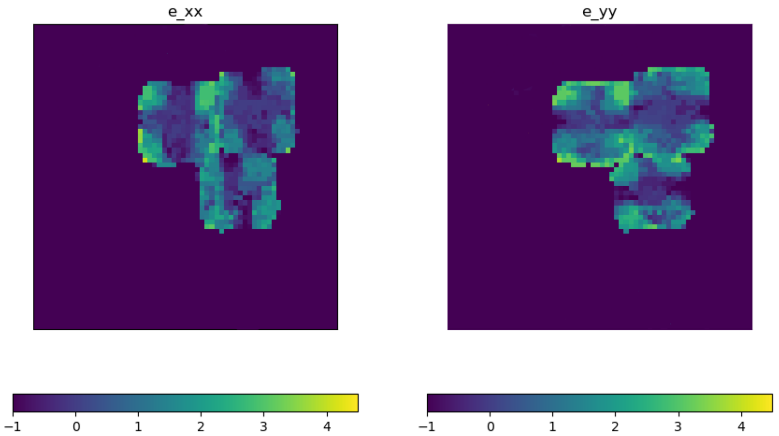

Supporting Figure S9. *Strain in 67 nm Pd nanoparticles measured at 160 °C on SiN in vacuum.*

5.2. General aspects of strain distribution in metal hydrogen systems

As long as the interface of metal and hydride is coherent, there is a linear relation between the difference of the local hydrogen concentrations and strain. For a free Pd-H particle, the linear expansion amounts to $0.063 \cdot \Delta c_H$. Hence, the overall strain of the material depends on the volume fractions of both phases. The situation is more complex in a core-shell structure since this configuration gives rise to additional constraint conditions. We have targeted this aspect in Ref. [18,19].

Beyond this, the local hydrogen concentration will change with the local stress state, as strain and concentration yield a feedback loop minimizing the total free energy of the system [18,19]. This yields the well-known Gorsky effect [20] of strain-driven diffusion.

References

[1] T. F. Kelly, M. K. Miller, *Rev. Sci. Instrum.* **2007**, *78*, 031101.

[2] C. Izawa, S. Wagner, M. Martin, S. Weber, R. Pargeter, T. Michler, H. H. Uchida, A. Pundt, *J. Alloys Compd.* **2013**, *580*, S13.

[3] K. Peeper, M. Moser, P. Reichart, E. Markina, M. Mayer, S. Lindig, M. Balden, G. Dollinger, , *J. Nucl. Mater.* **2013**, *438*, S887.

[4] S. Wagner, M. Moser, C. Greubel, K. Peeper, P. Reichart, A. Pundt, G. Dollinger, *Int. J. Hydrogen Energy* **2013**, *38*, 13822.

[5] D. Rohleder, P. Vana, *RSC Adv.* **2020**, *10*, 26504.

[6] W. Niu, L. Zhang, G. Xu, *ACS Nano* **2010**, *4*, 1987.

[7] R. F. Egerton, *Electron Energy-Loss Spectroscopy in the Electron Microscope*, Springer US, **2011**.

[8] U. Hohenester, *Comput. Phys. Commun.* **2014**, *185*, 1177.

[9] V. M. Silkin, R. Díez Muĩo, I. P. Chernov, E. V. Chulkov, P. M. Echenique, *J. Phys.: Condens. Matter* **2012**, *24*, 104021.

[10] F. J. García De Abajo, *Rev. Mod. Phys.* **2010**, *82*, 209.

[11] T. Tamaoka, H. Yoshida, S. Takeda, *RSC Adv.* **2019**, *9*, 9113.

[12] A. Baldi, T. C. Narayan, A. L. Koh, J. A. Dionne, *Nat. Mater.* **2014**, *13*, 1143.

[13] T. C. Narayan, A. Baldi, A. L. Koh, R. Sinclair, J. A. Dionne, *Nat. Mater.* **2016**, *15*, 768.

[14] J. Park, K. Bae, T. R. Kim, C. Perez, A. Sood, M. Asheghi, K. E. Goodson, W. Park, *Adv. Sci.* **2021**, *8*, 2002876.

[15] S. Korneychuk, B. Partoens, G. Guzzinati, R. Ramaneti, J. Derluyn, K. Haenen, J. Verbeeck, *Ultramicroscopy* **2018**, *189*, 76.

[16] A. Béché, J. L. Rouvière, L. Clément, J. M. Hartmann, *Appl. Phys. Lett.* **2009**, *95*, 123114.

[17] M. Nord, R. W. H. Webster, K. A. Paton, S. McVitie, D. McGrouther, I. MacLaren, G. W. Paterson, *Microsc. Microanal.* **2020**, *26*, 653.

[18] A. Dyck, T. Böhlke, A. Pundt, S. Wagner, *Scr. Mater.* **2024**, *247*, 116117.

[19] A. Dyck, J. Gisy, F. Hille, S. Wagner, A. Pundt, T. Böhlke, *submitted* **2024**.

[20] J. Völkl, *Berichte der Bunsengesellschaft für physikalische Chemie* **1972**, *76*, 797.
